# The New Surface Landmarks for Blind Axillary Vein Puncture

**DOI:** 10.21470/1678-9741-2019-0422

**Published:** 2020

**Authors:** Yaming Shi, Yongzhong Zong

**Affiliations:** 1 Department of Cardiology, Yancheng Third People’s Hospital, Yancheng, Jiangsu, People’s Republic of China.

**Keywords:** Axillary Vein, Vena Cava, Superior, X-Rays, Pneumothorax, Incidence, Control Groups, Crush Syndrome, Prospective Studies, Subclavian Vein, Pacemaker, Artificial

## Abstract

**Objective:**

To compare the efficacy of blind axillary vein puncture utilizing the new surface landmarks for the subclavian method.

**Methods:**

This prospective and randomized study was performed at two cardiology medical centers in East China. Five hundred thirty-eight patients indicated to undergo left-sided pacemaker or implantable cardioverter defibrillator implantation were enrolled, 272 patients under the axillary access and 266 patients under the subclavian approach. A new superficial landmark was used for the axillary venous approach, whereas conventional landmarks were used for the subclavian venous approach. We measured lead placement time and X-ray time from vein puncture until all leads were placed in superior vena cava. Meanwhile, the rate of success of lead placement and the type and incidence of complications were compared between the two groups.

**Results:**

There were no significant differences between the two groups in baseline characteristics or number of leads implanted. There were high success rates for both strategies (98.6% [494/501] *vs*. 98.4% [479/487], *P*=0.752) and similar complication rates (14% [38/272] *vs*. 15% [40/266], *P*=0.702). Six cases in the control group developed subclavian venous crush syndrome and five had pneumothorax, while neither pneumothorax nor subclavian venous crush syndrome was observed in the experimental group.

**Conclusion:**

We have developed a new blind approach to cannulate the axillary vein, which is as effective as the subclavian access, safer than that, and also allows to get this vein without the guidance of fluoroscopy, contrast, or echography.

**Table:** 

Abbreviations, acronyms & symbols	
**AMI** **AVB** **CRT** **DCM** **ICD** **ICM** **LBBB** **SPSS** **SSS** **SVC** **VT**	**= Acute myocardial infarction** **= Atrioventricular block** **= Cardiac resynchronization therapy** **= Dilated cardiomyopathy** **= Implantable cardioverter defibrillator** **= Ischemic cardiomyopathy** **= Left bundle branch block** **= Statistical Package for the Social Sciences** **= Sick sinus syndrome** **= Superior vena cava** **= Ventricular tachycardia**

## INTRODUCTION

Central venous access is an essential step during pacemaker and implantable cardioverter defibrillator (ICD) leads implantation^[1]^. Since first described in the late 1960s, the subclavian approach has emerged as the most frequently performed method for implanting endocardial pacemaker and transvenous defibrillator leads^[2]^. However, a 1% to 3% incidence of pneumothorax or hemothorax has been reported in association with the subclavian access^[3-5]^. Additionally, the subclavian access may result in an increased incidence of lead fracture due to entrapment of the lead by the costoclavicular ligament and/or the subclavius muscle^[6,7]^. Although blind axillary venous access was proved to be safe by Belott^[8]^, many physicians still cannulate central veins under tools guidance. The tools to facilitate cannulation, such as echography, are not available everywhere. Additionally, an expert in device implantation should master every option so he or she can choose the one that fits better in every situation.

## METHODS

From January 2012 to June 2014, all patients who presented to the cardiology department of the Yancheng Third People’s Hospital and the Nanjing Gulou Hospital in East China with indication to undergo left-sided pacemaker or ICD implantation were included in this study. The patients with indication for right-sided pacemaker or ICD implantation were excluded from this study. Any patient with indication for endocardial lead removal or upgrade was also excluded. Additionally, any patient who did not agree to participate in the study was excluded. All procedures followed our institutional guidelines and all patients provided their written informed consent. Patients’ demographics and clinical characteristics were recorded at baseline. Appling a simple randomized method, all patients were divided into experimental group and control group to receive implantation of endocardial electrode leads through axillary vein and subclavian vein approaches, respectively. The Research Ethics Committee of Yancheng Third People’s Hospital approved the study protocol (report of the ethics committee 2011-007).

Left subclavian venipuncture was performed through the skin at the level of the costoclavicular ligament using a G18 puncture needle attached to extension tubing and 10 ml syringe. The needle was inserted under negative pressure into the vein, 1-2 cm away from the external-inferior part of 1/3 point of intersection of medial-middle clavicle. The needle has clung closely to the skin or formed an angle of 30° with the skin, with the needle tip pointing to the sternal fovea superior or the Adam’s apple. The withdrawal of venous blood confirmed that the needle had entered the vein; afterwards, a standard 50-cm 0.038-inch J-tipped guide wire was then advanced through the needle to the superior vena cava (SVC), which was verified by X-ray fluoroscopy. A subcutaneous generator pocket was then created by an incision parallel to and approximately 2 cm below the clavicle.

**Fig. 1:**
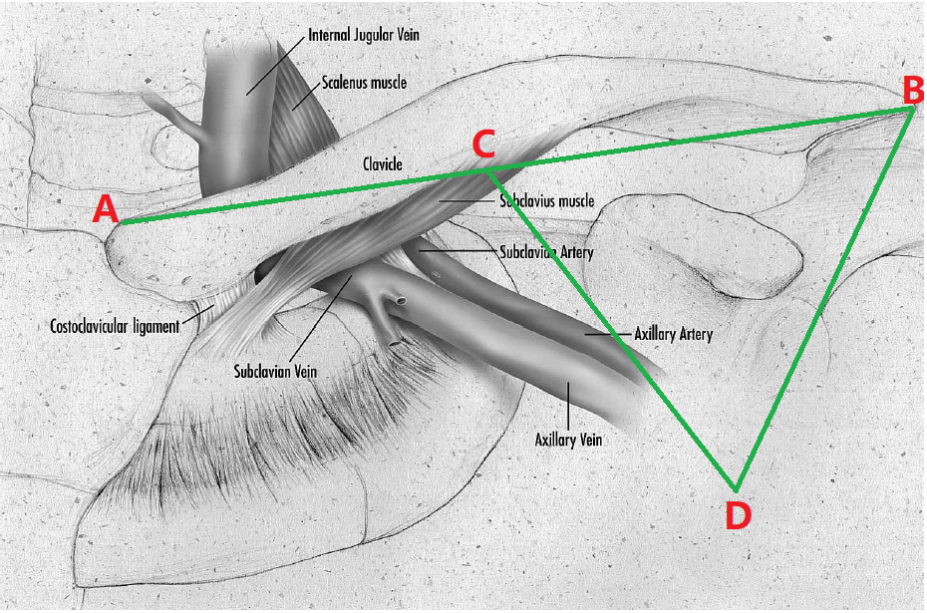
Diagram highlighting the access obtained by the axillary vein approach. The midpoint of the left clavicle (point C) was determined based on the position of both ends of the left clavicle (points A and B), and the connection between the middle point of the left clavicle and the acromion (line of the segment BC) was used as the bottom margin of the regular triangle; the vertex of the inverted regular triangle (point D) was determined as the puncture point. The G18 puncture needle is positioned at a 60° angle to the plane of the skin and a 60° angle to the bottom margin of the regular triangle. This diagram was modified from Ramza BM, et al.[5].

**Fig. 2:**
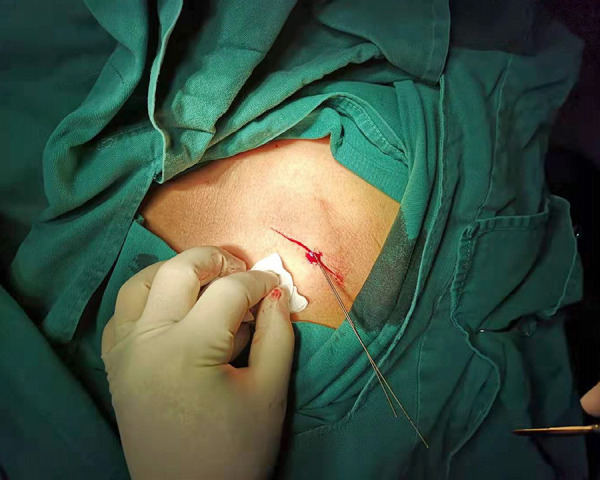
A subcutaneous generator pocket was created by an incision through the puncture point.

**Fig. 3:**
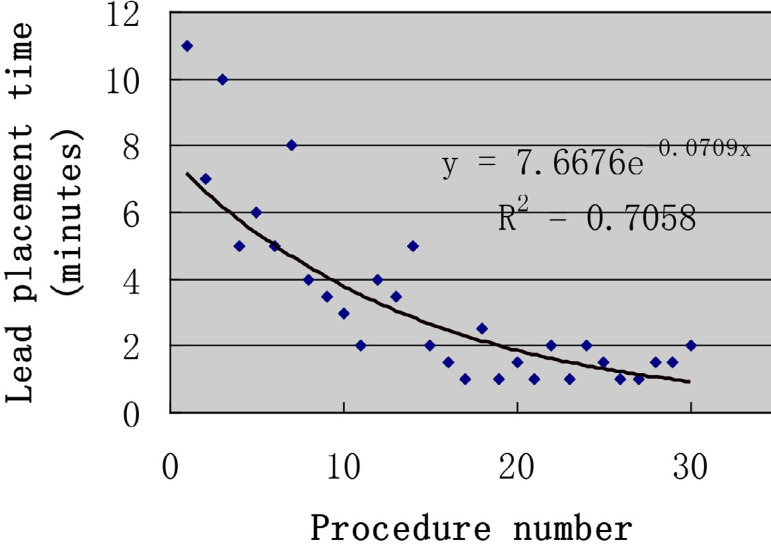
Time to place the leads in the superior vena cava for Operator 1.

**Fig. 4:**
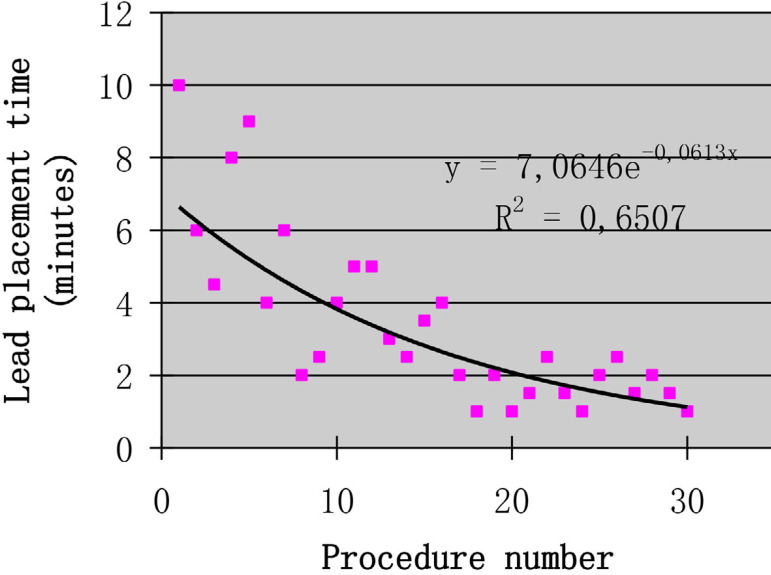
Time to place the leads in the superior vena cava for Operator 2.

The puncture method of the left axillary vein was as follows. Firstly, the midpoint of the left clavicle (point C) was determined based on the position of both ends of the left clavicle (points A and B). Secondly, the connection between the middle point of left clavicle and the acromion (line of the segment BC) was used as the bottom margin of the regular triangle, and the vertex of the inverted regular triangle (point D) was determined as the puncture point (Figure 1). Thirdly, the left axillary vein approach was performed through the skin, the G18 puncture needle was positioned at a 60° angle to the plane of the skin and a 60° angle to the bottom margin of the regular triangle. The needle was then advanced under suction until the vein was entered, as shown by a flash of blood in the syringe. If unsuccessful, the needle was moved either medially or laterally and the maneuver was repeated until the vein was entered. A standard 50-cm 0.038-inch J-tipped guide wire was then advanced through the needle to the SVC, which was verified by X-ray fluoroscopy. Separate punctures were used for each lead. A subcutaneous generator pocket was then created by an incision through the puncture point (Figure 2). At least five unsuccessful punctures were necessary to define a failure of the axillary vein access. In case of failure of the axillary vein access, the subclavian vein or the cephalic vein was sought. The vein puncture was carried out by two implanters. Prior to this study, learning of a blind axillary vein puncture technique was carried out by two implanters. According to the learning curve theory, axillary vein puncture training was completed when procedural times of two implanters was stabile^[9]^. Within three months, two operators completed 30 cases (Figures 3 and 4).

Lead placement time was measured from vein puncture until the fluoroscopic visualization of all leads in the SVC, and X-ray time was the time the fluoroscopic fluid used to reach this endpoint. The safety and effectiveness of the new surface landmarks for blind axillary vein puncture described in this study were evaluated by comparing the rate of success of lead placement and the type and incidence of complications. Chest X-ray film was obtained immediately after the procedure and in the following day to rule out evidence of pneumothorax and to check lead location. All participants were regularly followed up seven days, one month, and three months after the procedure, and then at intervals of one year at the device clinic in two hospitals. At each follow-up visit, generator and lead statuses, including fractures, insulation defects, and infections, subclavian or axillary vein thrombosis, and sensing and pacing parameters were examined.

Numerical variables are presented as median and standard deviation. Data were compared using Students’ paired *t*-test. Categorical variables, expressed as numbers and percentages, were compared using Chi-square or Fisher’s exact test. All statistical analyses were performed using the Statistical Package for the Social Sciences (SPSS) software (SPSS Inc, Chicago, Illinois, United States of America), version 13.0.

Patient and Public Involvement

This research project was constructed without patient involvement. Patients were not invited to comment on the study design and were not consulted to develop patient relevant outcomes or to interpret the results. Patients were not invited to contribute to the writing or editing of this document for readability or accuracy.

## RESULTS

There were 272 cases in the experimental group, including 176 males and 96 females, with age of 71.8±9.5 years; meanwhile, 266 cases were enrolled into the control group, including 171 males and 95 females, with age of 72.7±7.9 years. Differences in sex and age between the two groups were not statistically significant (*P*>0.05). The indication for pacemaker implantation was sick sinus syndrome in 238 patients and third-degree atrioventricular block in 176 patients. Of the 51 patients undergoing cardiac resynchronization therapy implantation, 28 had dilated cardiomyopathy with complete left bundle branch block (LBBB) and 33 had ischemic cardiomyopathy (ICM) with complete LBBB. Of the 73 patients who underwent ICD implantation, 32 had ventricular tachycardia (VT) 40 days after acute myocardial infarction and 41 had non-ICM complicated with VT. Five hundred and one endocardial electrode leads were implanted through the axillary vein approach in the experimental group, and 487 endocardial electrode leads were implanted through the subclavian vein approach. The difference in the disease type between the two groups was not statistically significant (*P*>0.05), as shown in Table 1. There were no differences of lead placement time and X-ray time between the two groups (2.30±1.93 minutes *vs.* 2.08±1.11 minutes and 51.3±10.4 seconds *vs.* 54.6±11.7 seconds, respectively). The successful puncture rates in the experimental and control groups were 98.6% (494/501) and 98.4% (479/487), respectively. Among the four cases with unsuccessful puncture in the experimental group, three were changed to implantation of endocardial electrode leads through the ipsilateral subclavian venipuncture, while one was changed to implantation of endocardial electrode leads through the contralateral subclavian venipuncture. All five cases with unsuccessful puncture in the control group were changed to implantation of endocardial electrode leads through the contralateral subclavian vein. Five cases in the control group had pneumothorax after surgery, and six had endocardial electrode lead rupture due to subclavian vein crush syndrome. One case in the experimental group had right ventricular electrode perforation and one had SVC syndrome. Seven cases in the experimental group and eight in the control group suffered from electrode dislocation, which was adjusted again through the original vein approach. Additionally, 10 cases in the control group and 12 in the experimental group had pouch hematomas, which subsided after pressure bandaging. Moreover, eight cases in the experimental group and three in the control group had left upper extremity venous thrombosis; among them, two in the experimental group improved after oral administration of warfarin and another three recovered after subcutaneous injection of low molecular weight heparin, while the remaining three in the experimental group as well as three in the control group recovered spontaneously two weeks after surgery by means of left upper extremity activity to promote the construction of collateral circulation. Comparisons between the results of both groups were shown in Table 2.

**Table 1:** Basic characteristics of patients in the two venous access groups.

Grouping category	Experimental group, n (%)	Control group, n (%)	*P*-value
Number of patients	272	266	
SSS	127 (46.7)	111 (41.7)	0.247
AVB	85 (31.3)	91 (34.7)	0.464
DCM with LBBB	13 (4.8)	15 (5.6)	0.654
ICM with LBBB	11 (4.0)	12 (4.5)	0.789
AMI with VT	17 (6.5)	15 (5.6)	0.765
ICM with VT	19 (7.0)	22 (8.3)	0.574

There were non-significant differences between the groups AMI=acute myocardial infarction; AVB=atrioventricular block; DCM=dilated cardiomyopathy; ICM=ischemic cardiomyopathy; LBBB=left bundle branch block; SSS=sick sinus syndrome; VT=ventricular tachycardia

**Table 2:** Comparison between the results of the two venous access groups.

Grouping category	Experimental group, n (%)	Control group, n (%)	*P*-value
Number of patients	272	266	
Lead placement time (minutes)	2.30±1.93	2.08±1.11	0.109
X-ray time (seconds)	51.3±10.4	54.6±11.7	0.327
Successful puncture rate	494/501 (98.6)	479/487 (98.4)	0.752
Pouch hematomas	12 (4.4)	10 (3.8)	0.702
Left upper extremity venous thrombosis	8 (2.9)	3 (1.1)	0.137
Pouch infection	9 (3.3)	8 (3.0)	0.770
Electrode dislocation	7 (2.6)	8 (3.0)	0.754
Pneumothorax	0 (0.0)	5 (1.9)	0.029
Subclavian vein crush syndrome	0 (0.0)	6 (2.3)	0.014
Right ventricular electrode perforation	1 (0.4)	0 (0.0)	0.322
Superior vena cava syndrome	1 (0.4)	0 (0.0)	0.322
Global complications	38 (14.0)	40 (15.0)	0.725

## DISCUSSION

Central venous access is an essential step during pacemaker and ICD leads implantation. The main approaches are subclavian, axillary, and cephalic accesses. Among those, the subclavian approach is the more frequently performed^[10]^, although it carries several risks, mainly lead fracture because of entrapment with the surrounding tissues (as high as 7%, according to Migliore et al.^[10]^, and a higher incidence of pneumothorax (1-3% *vs.* 0-1% of the axillary approach^[10-11]^. Cephalic vein access is very safe for both the electrode and the patient^[12]^, however it is more time consuming and technically challenging, especially when more than one electrode are to be implanted. On the other hand, the axillary approach combines both safety and a high success rate. Compared with the subclavian vein approach, the axillary venipuncture approach is associated with the following advantages. Firstly, the axillary venipuncture approach is not likely to induce crush syndrome due to the great distance when the endocardial electrode passes through the clavicle and the first intercostal spaces^[13]^. Secondly, the axillary vein runs outside the thoracic projection, and the puncture needle can form a certain angle with the thoracic wall, which is not likely to induce pneumothorax. Thirdly, the axillary vein is far away from the continued posterior artery and the subclavian vein, which can reduce the possibility of mistaken arterial puncture.

Blind axillary venous access was described several years ago^[8]^, but it is rarely used in a blind manner because of safety concerns^[14]^, although only one pneumothorax has been described by Belott^[15]^ after 168 implanted electrodes (0.6%). Nowadays, the axillary vein is canalized mainly with fluoroscopic or ultrasonic guidance^[9,16-18]^. However, these methods are time consuming in operation and will increase the costs, which are not appropriate for promotion and can only be used in some special conditions, such as venous malformation and failure in repeated puncture. Moreover, the body surface positioning methods described in the literature are complicated, which can hardly be mastered in practical application^[8,19]^. In this study, the three 60° axillary vein positioning and puncture method was adopted with the successful puncture rate of up to 98.6%, similar to the results of relevant studies^[20]^. Also in this study, six cases in the control group developed subclavian vein crush syndrome and five had pneumothorax, while none in the experimental group had pneumothorax or subclavian vein crush syndrome. The incidence of left upper extremity venous thrombosis was higher through the axillary vein approach than through the left subclavian vein approach, which was another noteworthy phenomenon in this study. Such phenomenon might be because the construction of collateral circulation of the axillary vein was relatively slow in the distal subclavian vein after thrombosis. Therefore, the patients should be closely observed after surgery and the left upper extremity activity in patients was encouraged to promote the construction of collateral circulation, which may be reduced the risk of venous thrombosis. Although the difference of left upper extremity venous thrombosis between two groups was not statistically significant, it may be due to the bias caused by the small sample size. Multicenter, randomized, double-blind larger sample studies are required for confirmation of the outcomes regarding the true efficacy of this blind approach to cannulate the axillary vein.

## CONCLUSION

We have developed a new blind approach to cannulate the axillary vein, which is as effective as the subclavian access, safer, and also allows to get this vein without the guidance of fluoroscopy, contrast, or echography. The benefits can be extended to emergency units for temporary pacemaker insertion as well as to intensive care units and pediatric units for central venous cannulation.

**Table:** 

Authors' roles & responsibilities	
YS YZ	Design of the study; final approval of the version to be published Acquisition of data; drafting the work; final approval of the version to be published
